# Effects of Different Chemicals on Sexual Regulation in Persimmon (*Diospyros kaki* Thunb.) Flowers

**DOI:** 10.3389/fpls.2022.876086

**Published:** 2022-05-26

**Authors:** Liyuan Wang, Huawei Li, Yujing Suo, Weijuan Han, Songfeng Diao, Yini Mai, Yiru Wang, Jiaying Yuan, Lingshuai Ye, Tingting Pu, Qi Zhang, Peng Sun, Fangdong Li, Jianmin Fu

**Affiliations:** Key Laboratory of Non-timber Forest Germplasm Enhancement & Utilization of State Administration of Forestry and Grassland, Research Institute of Non-timber Forestry, Chinese Academy of Forestry, Zhengzhou, China

**Keywords:** *Diospyros kaki* Thunb., sex differentiation, transcriptome, metabolome, exogenous regulation

## Abstract

Research on crop sexuality is important for establishing systems for germplasm innovation and cultivating improved varieties. In this study, androecious persimmon trees were treated with various concentrations of ethrel (100, 500, and 1,000 mg/L) and zeatin (1, 5, and 10 mg/L) to investigate the morphological, physiological, and molecular characteristics of persimmon. Ethrel at 1,000 mg/L and zeatin at 10 mg/L both significantly reduced the stamen length and pollen grain diameter in androecious trees. Ethrel treatment also led to reduced stamen development with degenerated cellular contents; zeatin treatment promoted the development of arrested pistils *via* maintaining relatively normal mitochondrial morphology. Both treatments altered carbohydrate, amino acid, and endogenous phytohormone contents, as well as genes associated with hormone production and floral organ development. Thereafter, we explored the combined effects of four chemicals, including ethrel and zeatin, as well as zebularine and 5-azacytidine, both of which are DNA methylation inhibitors, on androecious persimmon flower development. Morphological comparisons showed that stamen length, pollen viability, and pollen grain diameter were significantly inhibited after combined treatment. Large numbers of genes involving in carbohydrate metabolic, mitogen-activated protein kinase (MAPK) signaling, and ribosome pathways, and metabolites including uridine monophosphate (UMP) and cyclamic acid were identified in response to the treatment, indicating complex regulatory mechanisms. An association analysis of transcriptomic and metabolomic data indicated that ribosomal genes have distinct effects on UMP and cyclamic acid metabolites, explaining how male floral buds of androecious persimmon trees respond to these exogenous chemicals. These findings extend the knowledge concerning sexual differentiation in persimmon; they also provide a theoretical basis for molecular breeding, high-yield cultivation, and quality improvement in persimmon.

## Introduction

*Diospyros kaki* Thunb. is an economically important fruit tree species in China (Luo and Wang, 2008), which can be classified into two types including pollination-constant non-astringent (PCNA) and non-PCNA. The PCNA type is divided into Japanese PCNA and Chinese PCNA subtypes, according to the regulatory mechanisms underlying natural deastringency in fruits. The non-PCNA type is divided into pollination-variant non-astringent, pollination-variant astringent, and pollination-constant astringent subtypes (Akagi et al., 2011; Xu et al., 2016). Persimmons can be categorized into four types according to the expression of sex characteristics at the individual level: (i) only female flowers (gynoecious-type), (ii) only male flowers (androecious-type), (iii) both male and female flowers (monoecious-type), and (iv) both bisexual and unisexual flowers (trimonoecious-type) (Yakushiji et al., 1995; Huang et al., 2013; Zhang et al., 2016; Guan et al., 2020). Female flowers are usually solitary with a central flower per inflorescence, along with two lateral aborted flowers; male flowers occur in cymose clusters of three to five flowers with fertile stamens and solitary arrested carpels (George et al., 1997; Akagi et al., 2014). The key point for the loss of inappropriate sex organs largely related to programmed cell death, occurs in mid-April (Wang L. Y. et al., 2020). Most domesticated persimmon cultivars are gynoecious and monoecious because of their strong parthenocarpic ability; the androecious types have been ignored by farmers. This situation restricts the breeding of new persimmon cultivars *via* cross-pollination. Hence, there is a need to establish techniques for new breeding systems to facilitate germplasm innovation and the cultivation of improved varieties. Among such techniques, artificial treatments that regulate sex differentiation of persimmon are useful from both theoretical and practical perspectives.

Recent studies have identified some sex determination genes in a small subset of plants. *CS-ACS2* and *CS-ACS1G* are sex-determining genes in cucumber (Saito et al., 2007), while *CmACS-7* and *CmWIP1* are sex-determining genes in melon (Martin et al., 2009). *NA1* (associated with brassinosteroid synthesis) and *TS1* (associated with jasmonic acid synthesis) were identified as sex-determining genes in monoecious maize (*Zea mays*) (Hartwig et al., 2011). Moreover, *SpGAI* functions as a feminizing factor in spinach (West and Golenberg, 2018); *ARR17* serves as a sex switch in poplar, triggering female development when on and male development when off (Müller et al., 2018). Genomics and transcriptomics analyses, combined with gene-editing and complementation analyses, led to the identification of two potential sex determinant genes in kiwifruits: *FrBy* (a dominant suppressor of carpel development) and *SyGI* (maintains male functions) (Akagi et al., 2018a,b). Massonnet et al. (2020) identified two candidate genes (*VviINP1* and *VviYABBY3*) associated with male-sterility mutation and female sterility by comparing twenty *Vitis* SDR haplotypes with gene expression data. Comparisons of contiguous X and Y chromosome assemblies showed that the *SOFF* and *aspTDF1* genes suppress female organogenesis and promote male function, respectively (Harkess et al., 2020). According to Akagi et al. (2016), sexuality in persimmon is controlled by the methylation level of a promoter of the sex determination gene *MeGI*; a high level of DNA methylation in the *MeGI* promoter leads to the development of male flowers. Notably, DNA methylation is a mechanism that underlies epigenetic regulation; it could affect gene regulation and phytohormone homeostasis (Mateo-Bonmatí et al., 2019). Consistent with this hypothesis, the foliar application of 40 mg/L 5-azacytidine (a DNA methylation inhibitor) was effective for disrupting bud dormancy *via* DNA demethylation and the reduction of abscisic acid (ABA) levels (Kondo et al., 2010; Zhou et al., 2016). In addition, Akagi et al. (2016) investigated the effect of the non-specific inhibitor zebularine on sex determination in persimmon; they found that treatment with 1 mM exogenous zebularine inhibited anther development and induced developing male buds to form feminized flowers.

Phytohormones are important regulators of sex differentiation in many plants. In particular, ethylene has a major role in female-specific gene expression in different species (Martin et al., 2009; Pawełkowicz et al., 2019). We previously showed that zeatin (ZT) levels were much higher in female flower buds than in male flower buds from early April to early May, which implies that high levels of ZT may promote the development of female floral buds (Sun et al., 2017). In addition, RNA sequencing and comparative analysis between male and female floral buds of persimmon revealed that a gene homologous to *GA20OX2* (an indicator of gibberellin [GA] biosynthetic enzymes) (Plackett et al., 2011) may stimulate the development of male floral buds in April. By contrast, a gene homologous to *ACO* (a promoter of ethylene biosynthesis) (Trivellini et al., 2011) showed female-promoting effects from early April to early May (Li et al., 2019). Based on the above findings, we injected ethrel (100 mg/L) into androecious wild persimmon trees; we demonstrated that ethrel treatment markedly decreased the pollen grain diameter and the pollen tube length in androecious persimmon flowers, highlighting its inhibitory effect on androecia development (Wang et al., 2021). Studies regarding sexual differentiation of persimmon have made considerable progress. However, our previous study focused on the effect of a single concentration of ethrel on persimmon sex differentiation; there remains minimal information regarding the combined effects of a mixture of zebularine, 5-azacytidine, ethrel, and ZT on persimmon flower development. In addition, studies of persimmon abiotic stress responses have mainly focused on physiological and biochemical aspects, rather than molecular response mechanisms. Thus, flower development candidate genes and the metabolite response to plant growth regulators in persimmon remain unclear; these aspects should be explored using metabolomics and transcriptomics analyses.

In this study, we conducted a comprehensive analysis of floral development during exposure to distinct exogenous phytohormone conditions. The study objectives were to (1) identify ZT and ethrel treatments for floral development and sex differentiation, including the phenotypic traits, gene expression profiles, carbohydrate metabolism dynamics, nutrient elements, amino acid levels, and endogenous hormone concentrations; (2) comprehensively explore the molecular mechanisms active in androecious persimmon at transcriptome and metabolome levels, while seeking links between genes and metabolites in response to combined treatment with a mixture of four chemicals (zebularine, 5-azacytidine, ethrel, and ZT); and (3) construct an overall schematic of the response to artificial treatment that could be useful for future efforts to control sex type in persimmon.

## Materials and Methods

### Plant Materials

Experiments were conducted using two types of 5-year-old androecious persimmon trees, “Mulan Yeshi” and “Yunjiashan Yeshi,” both of which originated from Hubei Province, China, and have a close genetic relationship (Wang et al., 2018). These trees were grown in pots in the Persimmon Germplasm Repository (Yuanyang County, Henan Province, China; 34°55.30’–34°56.45’N, 113°46.24’–113°47.59E), which is owned by the Research Institute of Non-timber Forestry, Chinese Academy of Forestry.

### Treatments

Various concentrations of ethrel and ZT were used for foliar application (Table 1). Each treatment was applied to five replicate trees; applications were conducted once weekly from March 22, 2020 (immediately after bud burst and stamen primordia initiation) through May 3, 2020 (prior to anthesis). The control treatment comprised trees that did not undergo foliar application of plant growth regulators. Approximately 20 g of male flowers were collected from each tree on May 6, 2020 (at anthesis and mature pollen/embryo sac formation) for morphological, physiological, and molecular (i.e., real-time quantitative polymerase chain reaction [RT-qPCR]) analyses.

**TABLE 1:** Treatments and types of treated trees.

Growth regulator	Concentration	Sex type of treated trees
Ethrel	100 mg/L	Androecious “Mulan Yeshi”
Ethrel	500 mg/L	Androecious “Mulan Yeshi”
Ethrel	1000 mg/L	Androecious “Mulan Yeshi”
ZT	1 mg/L	Androecious “Mulan Yeshi”
ZT	5 mg/L	Androecious “Mulan Yeshi”
ZT	10 mg/L	Androecious “Mulan Yeshi”

Next, “Yunjiashan Yeshi” persimmon received combined applications of the following four regulators: 1 mM zebularine, 0.2 mM 5-azacytidine, 2 g/L ethrel, and 20 mg/L ZT. The foliar treatment was applied to five replicate trees on May 3, 2021. Foliar application of water was used as the control treatment. Approximately 20 g of male flowers were collected from each tree on May 6 for morphological, transcriptomics, and metabolomics analyses.

### Phenotypic Determination and Observation

Lengths of stamens and pollen tubes, diameters of pollen grains, and pollen viability in androecious trees were assessed using the methods of Wang et al. (2021). Hematoxylin and eosin staining and transmission electron microscopy observations were conducted using the methods of Wang L. Y. et al. (2020).

The pollen surface was imaged at high resolution using scanning electron microscopy (Carl Zeiss-EvoLS 10). Samples were fixed overnight using 2.5% glutaraldehyde in 0.1 M potassium phosphate buffer. After three washes with phosphate-buffered saline (pH = 7.4) for 10 min, the samples were dehydrated for 20 min in a graded ethanol series (30, 50, 70, and 90%). The pollens were critical point-dried using CO_2_, then mounted on spherical metal stubs and fixed using adhesive tape. Finally, the samples were shadowed with gold in a sputter coater instrument and viewed using a scanning electron microscope.

### Measurement of Physiological Indices

The carbohydrate, phosphorus (P), and potassium (K) contents were assayed using test kits from the Nanjing Jiancheng Bioengineering Institute (Jiangsu, China), in accordance with the manufacturer’s instructions. Nitrogen (N) contents were determined using a KJELTEDC AUTO-2300 automatic nitrogen analyzer with 0.5 g of sample that had been digested in 5 mL concentrated sulfuric acid at 280°C. Soluble proteins were measured using the Coomassie Brilliant Blue G-250 method. Amino acid contents were measured using an amino acid analyzer (Hitachi, Japan). Endogenous phytohormone levels were quantified by high-performance liquid chromatography/electrospray ionization tandem mass spectrometry, using the method of Sun et al. (2017).

### Transcriptome Assembly and Differentially Expressed Gene Annotation and Identification

To identify genes associated with stamen development, three biological replicates each were selected from the non-treated and treated groups for cDNA library construction and transcriptome sequencing using the DNBSEQ-T7 platform (BGI Group, Shenzhen, Guangdong, China).

Total stamen RNA was isolated using TRIzol Reagent (Invitrogen, Carlsbad, CA, United States) and detected on a 1% agarose gel. RNA concentrations and RNA integrity numbers were assessed using a NanoDrop 2000 spectrophotometer (Thermo Fisher Scientific, MA, United States) at 260/280 nm (ratio > 2.0) and an Agilent 2100 bioanalyzer (Agilent, Santa Clara, CA, United States). The TruSeq RNA Sample Preparation Kit (Illumina, San Diego, CA, United States) was used for sequencing library construction, in accordance with the manufacturer’s recommendations. After mRNA enrichment and purification, the library was sequenced.

After the removal of low-quality reads and reads containing adaptors and poly N sequences, clean reads were obtained. The clean reads were assembled using Trinity software, version 2.0.6^1^. Fragments per kilobase of exon per million mapped fragments values were used to calculate gene expression levels. DEseq2^2^ was used to detect differentially expressed genes (DEGs) with the following criteria: (i) fold change ≥ 2 and (ii) *Q* value ≤ 0.05. Phyper was used to conduct Gene Ontology (GO) and Kyoto Encyclopedia of Genes and Genomes (KEGG) enrichment analyses.

### Metabolite Extraction, Liquid Chromatography/Mass Spectrometry Assignment, and Profiling

Cryopreserved biomaterial stamens (25 mg per sample) were weighed and placed into 1.5 mL tubes containing methanol/acetonitrile/water (2:2:1, vol/vol/vol). A Waters 2D UPLC (Waters, Milford, MA, United States) coupled with ultra-high performance liquid chromatography (Thermo Fisher Scientific) was used for metabolic profiling analysis. Samples (5 μL injection volumes) were injected onto a BEH C18 column (1.7 μm, 2.1*100 mm, Waters) operating at 40°C; a flow rate of 0.3 mL/min was used for positive and negative modes. Compounds were separated using the linear elution gradient: 98:2 Phase A/Phase B at 0–1 min; 98:2–2:98 Phase A/Phase B at 1–9 min; 2:98 Phase A/Phase B at 9–12 min; 2:98–98:2 Phase A/Phase B at 12–12.1 min; and 98:2 Phase A/Phase B at 12.1–15 min. Mass spectrometry data were measured using a Q Exactive spectrometer (Thermo Fisher Scientific).

After mass spectrometry analysis, the raw data were converted into a common format using Compound Discoverer 3.0 (Thermo Fisher Scientific) for qualitative and relative quantitative analysis. Peak intensity, mass-to-charge ratio, and retention time were analyzed for peak integration and correction. Significantly changed metabolites (SCMs) among different comparison groups were identified using the following criteria: *P* < 0.05 (two-tailed Student’s test, normalized peak areas) and fold change ≥ 1.2 or ≤ 0.83.

Pearson correlation coefficients of DEGs and SCMs were calculated using the COR package in R; coefficients of > 0.99 or < −0.99 (with *P* < 0.001) were considered to indicate significant correlations. Coexpression analysis network diagrams were drawn using Cytoscape software, version 3.4.0.

### Real-Time Quantitative Polymerase Chain Reaction for Expression Analysis Following Ethrel and Zeatin Treatments

Total RNA was extracted using EZ-10 DNA-away RNA Mini-Preps (Sangon Biotech, Shanghai, China), in accordance with the manufacturer’s instructions. First-strand cDNA was synthesized using a TRUEscript 1st Strand cDNA Synthesis Kit (Kemix). All RT-qPCR experiments were performed with the 2 × Sybr qPCR Mix (Kemix) using a CFX96™ Real-Time System (Bio-Rad, Hercules, CA, United States) and the following protocol: denaturation at 94°C for 2 min, followed by 40 cycles of 94°C for 20 s, 55–60°C for 20 s, and 72°C for 30 s. The primers for genes associated with hormone production and floral organ development are listed in Supplementary Table 1 based on our published transcriptome sequencing protocol (Li et al., 2019). Each RT-qPCR reaction was performed with three biological replicates, and each sample was analyzed with three technical replicates, using *GAPDH* as an internal control.

### Real-Time Quantitative Polymerase Chain Reaction for RNA-Seq Validation

The expression levels of eight randomly selected genes were determined by RT-qPCR to validate the accuracy of the RNA-Seq data. This study was conducted using the same samples as those used for transcriptome sequencing. The procedures used for RNA extraction, cDNA synthesis, and RT-qPCR experiments were consistent with those described for ethrel and ZT treatment. Primers corresponding to the conserved region of each cDNA were designed based on the transcriptome sequencing data used in this study and are listed in Supplementary Table 2. Each RT-qPCR was performed with three biological replicates, and each sample was analyzed in triplicate.

### Statistical Analyses

The 2^–ΔΔ*CT*^ method was used to calculate relative gene expression levels from RT-qPCR data. All data with three replicate values are summarized here as means ± standard errors. One-way analysis of variance, executed using SPSS for Windows (ver. 20.0; SPSS, Inc., Chicago, IL, United States), was employed to identify significant differences among the treatment and control groups. Means were compared using Tukey’s test; *P* < 0.05 was considered to indicate statistical significance.

## Results

### Phenotypic Changes in Androecious Male Flowers After Treatment With Ethrel and Zeatin

**FIGURE 1:**
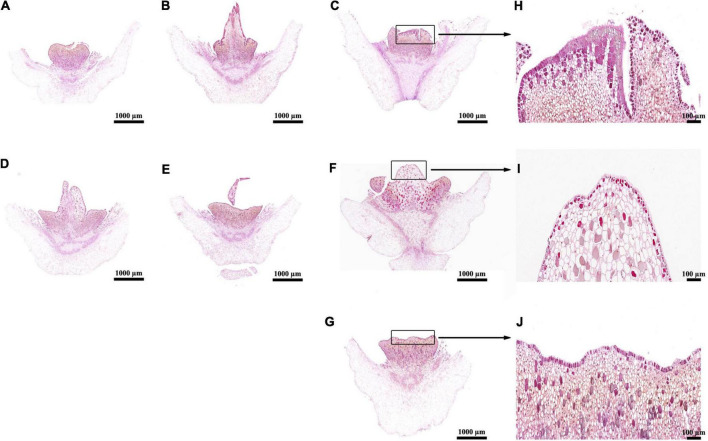
Histological analyses of aborted pistils in ethrel- and ZT-treated androecious persimmon flowers. **(A–G)** Samples treated with ethrel at 100, 500, and 1,000 mg/L; ZT at 1, 5, and 10 mg/L; and control (untreated). **(H)** Magnified image of the aborted pistil highlighted in **(C)**. **(I)** Magnified image of the aborted pistil highlighted in **(F)**. **(J)** Magnified image of the aborted pistil highlighted in **(G)**.

Applications of 500 and 1,000 mg/L ethrel, as well as 1–10 mg/L ZT, led to significantly shorter stamen lengths (Table 2). The pollen grain diameter also significantly decreased in plants treated with 100–1,000 mg/L ethrel, as well as 5 and 10 mg/L ZT. In addition, pollen viability was suppressed by treatment with 1,000 mg/L ethrel. Overall, 1,000 mg/L ethrel appeared to be optimal for modifying stamen development *via* robust reduction of stamen length, pollen grain diameter, and pollen viability, compared to the other treatments. Morphogenetic analysis of inappropriate sex organs revealed markedly larger aborted pistils in 10 mg/L ZT-treated androecious flowers, along with reduced cell density and enhanced cell size, compared to the other treatments (Figure 1). This observation indicated that 10 mg/L ZT treatment affected the regulation of cell patterns, thus altering aborted pistil phenotypes.

**TABLE 2:** Effects of exogenous ethrel and ZT on flower traits in androecious persimmon.

Treatment	Stamen length (mm)	Pollen tube length (μm)	Pollen grain diameter (μm)	Pollen viability (%)
Ethrel, 100 mg/L	7.78 ± 0.83 a	178.56 ± 17.88 ab	47.45 ± 3.10 b	45.47 ± 6.17 b
Ethrel, 500 mg/L	7.05 ± 0.86 b	189.92 ± 29.83 ab	47.23 ± 3.74 bc	32.54 ± 8.75 c
Ethrel, 1,000 mg/L	6.48 ± 0.93 c	175.56 ± 29.34 b	45.23 ± 3.81 d	29.81 ± 4.82 c
ZT, 1 mg/L	5.59 ± 0.86 e	209.65 ± 38.23 ab	49.33 ± 3.36 a	53.48 ± 9.35 a
ZT, 5 mg/L	5.63 ± 0.77 e	222.30 ± 40.62 a	46.49 ± 2.87 c	52.40 ± 10.75 a
ZT, 10 mg/L	6.05 ± 0.81 d	212.59 ± 39.37 ab	44.63 ± 3.06 d	53.07 ± 6.78 a
Control	7.64 ± 0.94 a	191.45 ± 35.21 ab	49.77 ± 4.20 a	30.20 ± 4.63 c

*Different letters denote significant differences among the treated and control groups (Tukey’s test, P < 0.05).*

**FIGURE 2:**
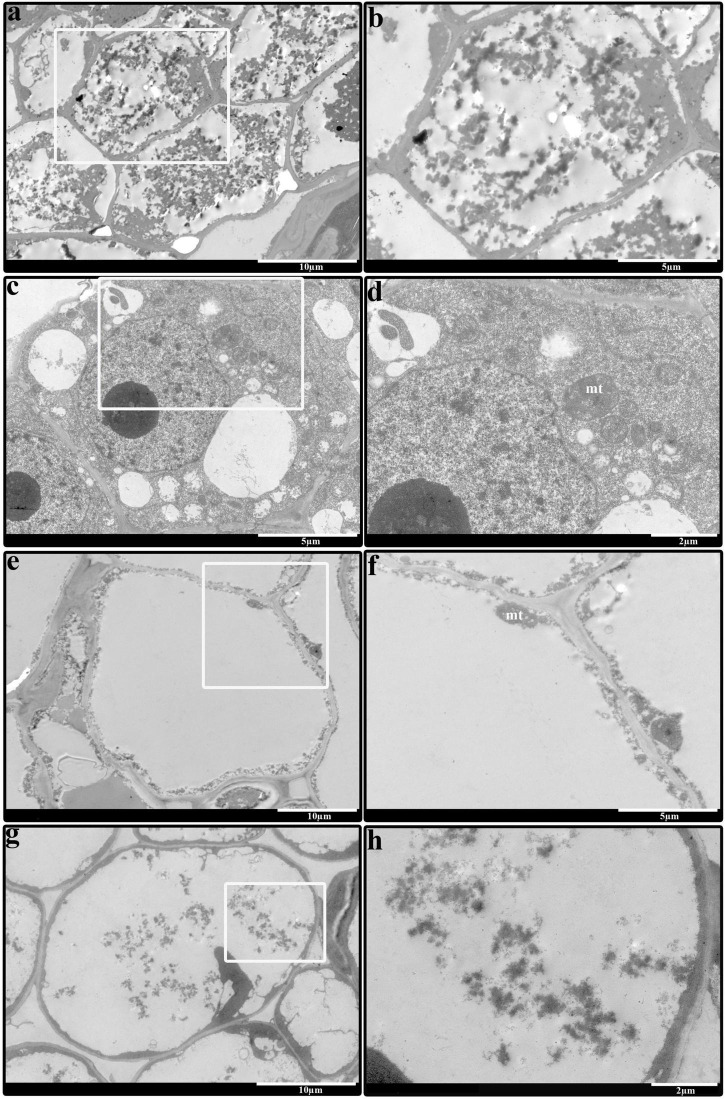
Comparison of stamen and aborted pistil in androecious persimmon flowers. **(a)** Electron microscopic structure of the stamen from a sample treated with 1,000 mg/L ethrel. **(b)** Magnified image highlighted in **(a)**. **(c)** Electron microscopic structure of the stamen from an untreated sample. **(d)** Magnified image highlighted in **(c)**. **(e)** Electron microscopic structure of the aborted pistil from a sample treated with 10 mg/L ZT. **(f)** Magnified image highlighted in **(e)**. **(g)** Electron microscopic structure of the aborted pistil from an untreated sample. **(h)** Magnified image highlighted in **(g)**. mt, mitochondria.

The above results showed that 1,000 mg/L ethrel had a significant effect on stamen morphology in androecious persimmon flowers, compared with untreated flowers; 10 mg/L ZT had a significant effect on aborted pistil morphology, compared with untreated flowers. Thus, we observed the electron microscopic structure of stamens and aborted pistils in flowers that had received the two treatments. Our studies revealed that the cellular contents in ethrel-treated stamen were degenerated (Figures 2a,b), moreover, their volume densities were reduced compared with the untreated stamen (Figures 2c,d). Transmission electron microscopy images showed that relatively normal mitochondrion was observed in the ZT-treated aborted pistil (Figures 2e,f) in comparison of untreated sample (Figures 2g,h).

### Physiological Indices and Gene Expression in Response to Exogenous Application of Ethrel and Zeatin

**FIGURE 3:**
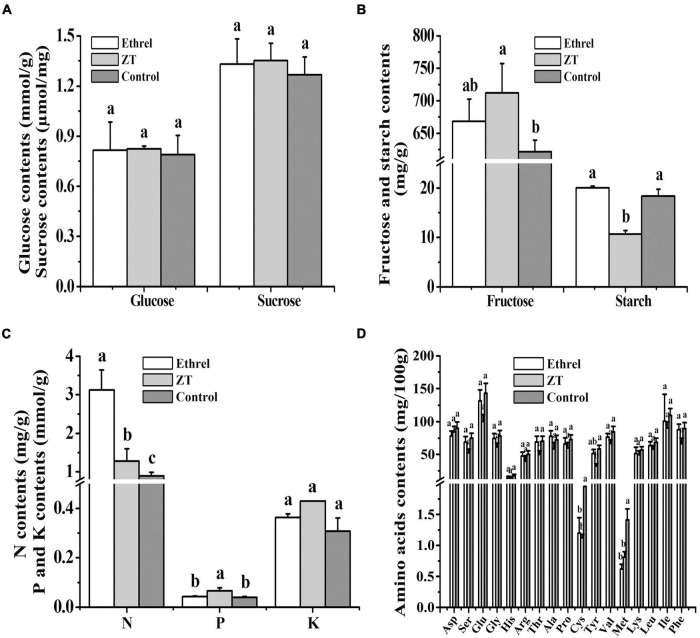
Effects of ethrel and ZT treatments on the contents of glucose and sucrose **(A)**; fructose and starch **(B)**; nitrogen, phosphorus, and potassium **(C)**; and amino acids **(D)**. Different letters denote significant differences among the treated and control groups (Tukey’s test, *P* < 0.05).

**FIGURE 4:**
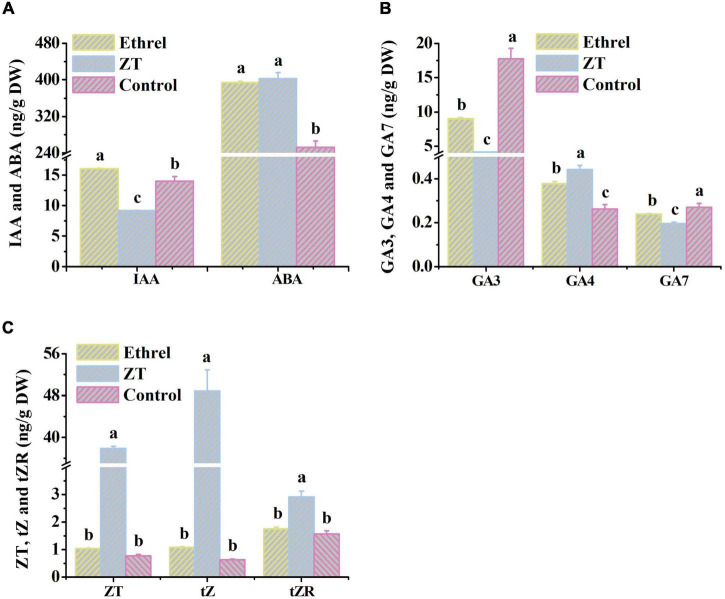
Changes in endogenous hormone levels in androecious persimmon flowers in response to ethrel and ZT treatment. **(A)** Contents of IAA and ABA. **(B)** Contents of GA_3_, GA_4_ and GA_7_. **(C)** Contents of ZT, tZ and tZR. Different letters denote significant differences among the treated and control groups (Tukey’s test, *P* < 0.05).

**FIGURE 5:**
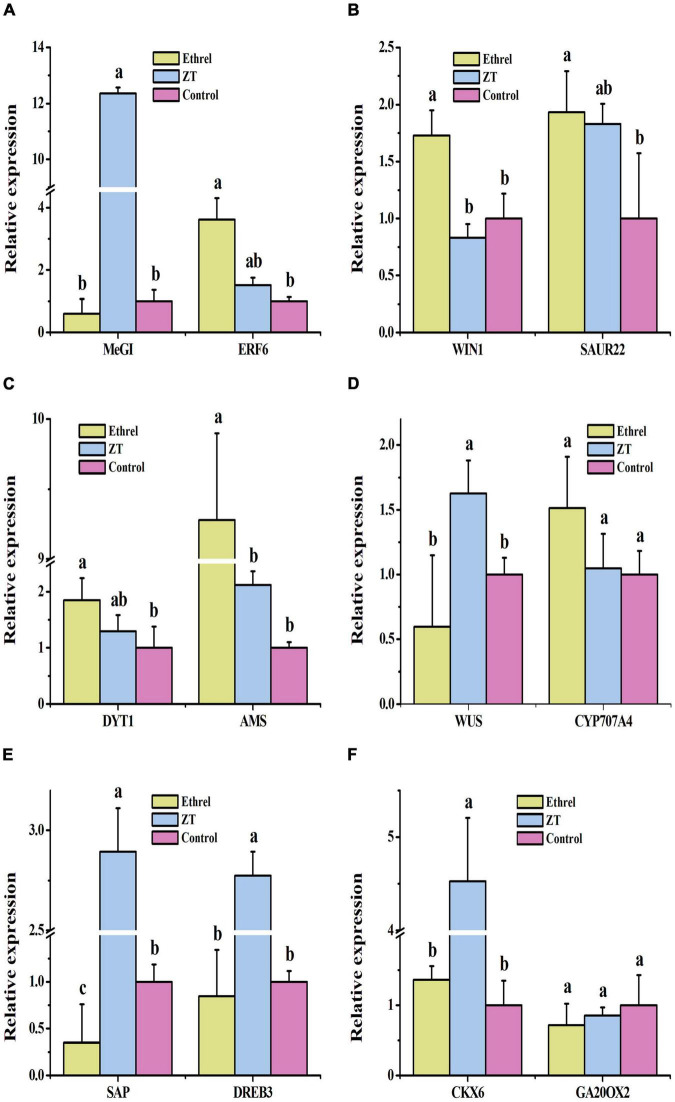
Effects of exogenous ethrel and ZT on the relative expression levels of candidate genes in androecious persimmon flowers. **(A)** Relative expression levels of *MeGI* and *ERF6*. **(B)** Relative expression levels of *WIN1* and *SAUR22*. **(C)** Relative expression levels of *DYT1* and *AMS*. **(D)** Relative expression levels of *WUS* and *CYP707A4*. **(E)** Relative expression levels of *SAP* and *DREB3*. **(F)** Relative expression levels of *CKX6* and *GA20OX2*. Different letters denote significant differences among the treated and control groups (Tukey’s test, *P* < 0.05).

Physiological indices and gene expression levels in androecious male flowers showed distinct changes after treatment with 1,000 mg/L ethrel, 10 mg/L ZT, and control (untreated). Overall, the application of ethrel induced high levels of indoleacetic acid (IAA) hormone, ethylene- and auxin-related genes (*WIN1*, *ERF6*, and *SAUR22*), and floral organ development-related genes (*DYT1* and *AMS*); it reduced the levels of ZT, *trans-*ZT (tZ), and *trans-*zeatin riboside (tZR) hormones, as well as the *SAP* gene. Furthermore, high levels of ABA, ZT, tZ, and tZR hormones; P and fructose substances; and cytokinin-, ABA-, and floral organ development-related genes (*CKX6*, *DREB3*, *MeGI*, *WUS*, and *SAP*) were observed after ZT treatment. These findings highlight the distinct effects of these plant growth regulators on persimmon flowers (Figures 3–5).

### Stamen and Pollen Development Were Inhibited by Combined Treatment

**FIGURE 6:**
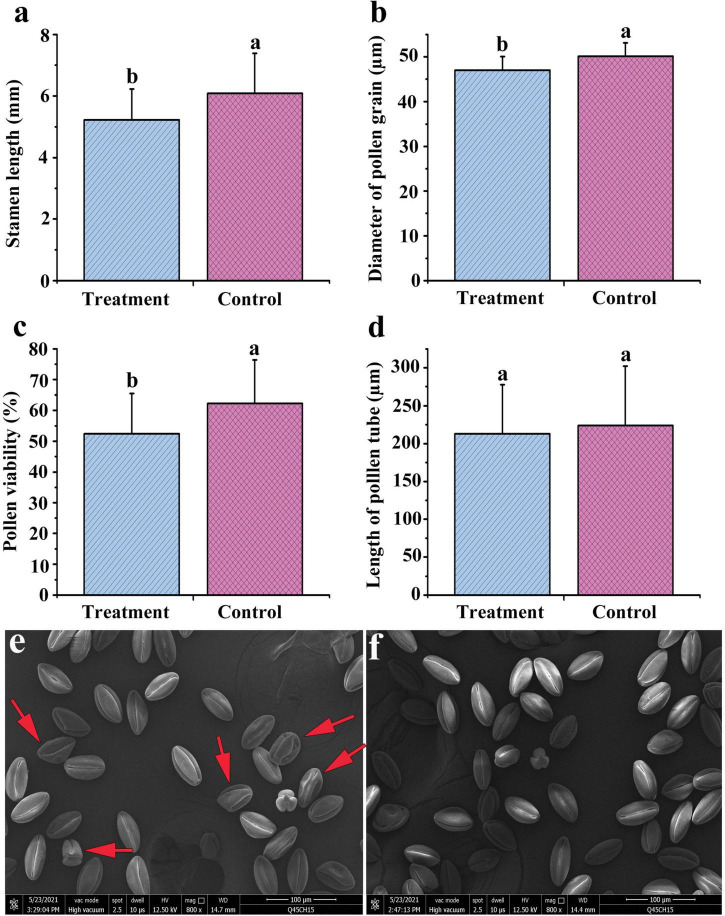
Stamen and pollen development in androecious “Yunjiashan Yeshi” trees, compared between control and combined treatment with exogenous zebularine, 5-azacytidine, ethrel, and ZT. **(a–d)** Comparison of phenotypic traits between treated and control samples. **(e,f)** Electron microscopy comparisons of pollen grains in treated and control samples.

We concluded that foliar spraying of ethrel at 1,000 mg/L and ZT at 10 mg/L significantly reduced stamen development and altered the carbohydrate, amino acid, and endogenous phytohormone contents, as well as genes associated with hormone production and floral organ development. Next, we further increased the treatment dosages and sprayed a combination of four chemicals, including ethrel at 2 g/L, ZT at 20 mg/L, zebularine at 1 mM, and 5-azacytidine at 0.2 mM, on male floral buds to investigate the morphological, transcriptomic, and metabolomic responses of male persimmon flowers. Zebularine and 5-azacytidine were reported to be DNA methylation inhibitors and showed female promoting effect (Akagi et al., 2016). Considering the changes in stamen and pollen development between treated and control androecious persimmon, we concluded that “Yunjiashan Yeshi” was sensitive to the mixture of zebularine, 5-azacytidine, ethrel, and ZT. Although the treatment did not affect pollen tube length, it considerably reduced stamen length, pollen viability, and pollen grain diameter (Figures 6a–d). In addition, morphological observation of pollen grains was performed by scanning electron microscopy to characterize the effects of the combined treatment on pollen grains. Scanning electron micrographs revealed many shriveled pollen grains with irregular morphology in the treated samples (Figures 6e,f).

### Transcriptome Sequencing of the Combined Treatment Response

In total, 266,987,806 clean reads were generated from six samples; each sample produced a minimum of 44.05 M clean reads, and the Q30 values were > 92.47%, indicating high sequencing quality (Supplementary Table 3). Among these clean reads, 70.77–77.18% were mapped to the *Diospyros oleifera* reference genome (NCBI, BioProject, PRJNA532832). After quality control processing, the clean reads of the six libraries were assembled into 26,274 unigenes with distinct fragments per kilobase of exon per million mapped fragments values (Supplementary Table 4). The RNA-Seq data sets were deposited in the China National Center for Bioinformation National Genomics Data Center (CNCB–NGDC) Genome Sequence Archive (GSA) database (accession no. CRA006019).

**FIGURE 7:**
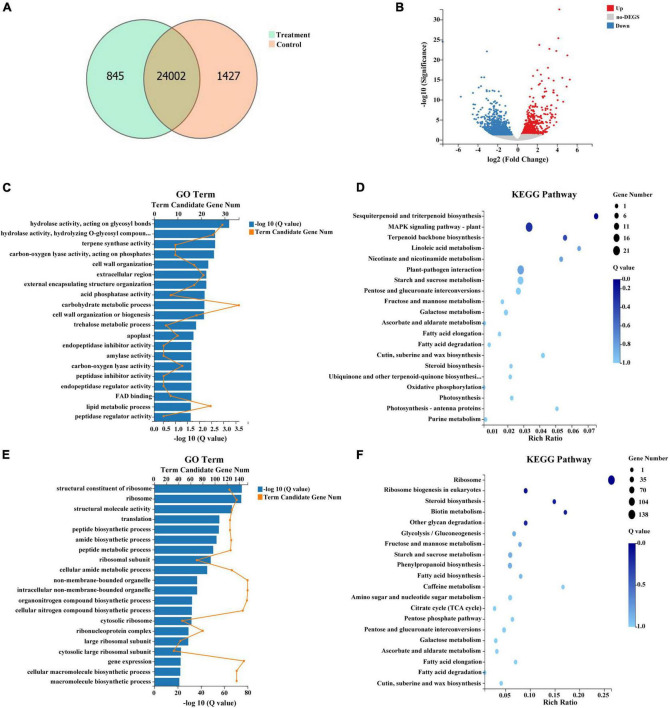
Summary of differentially expressed genes (DEGs) in response to combined treatment. **(A)** Venn diagram of DEGs from the transcriptome. **(B)** Volcano plot of DEGs from the transcriptome. **(C)** Gene ontology (GO) pathway analysis of upregulated DEGs. **(D)** Kyoto Encyclopedia of Genes and Genomes (KEGG) pathway analysis of upregulated DEGs. **(E)** GO pathway analysis of downregulated DEGs. **(F)** KEGG pathway analysis of downregulated DEGs.

In total, 24,847 and 25,429 genes were expressed in treatment and control groups, respectively (Figure 7A). Upon comparison of stamens in treatment and control groups, 1,539 genes demonstrated significant differential expression, including 478 genes that were upregulated in the treatment group and 1,061 genes that were downregulated in the treatment group (Figure 7B and Supplementary Table 5). A GO analysis of the DEGs between treatment and control groups revealed enrichment in 20 categories. The most highly enriched GO categories for the upregulated DEGs were carbohydrate metabolic process (GO:0005975; 36 unigenes); hydrolase activity, acting on glycosyl bonds (GO:0016798; 29 unigenes); and hydrolase activity, hydrolyzing O-glycosyl compounds (GO:0004553; 25 unigenes) (Figure 7C). The most highly enriched GO categories for the downregulated DEGs were non-membrane-bounded organelle (GO:0043228; 154 unigenes); intracellular non-membrane-bounded organelle (GO:0043232; 154 unigenes); and organonitrogen compound biosynthetic process (GO:1901566; 153 unigenes) (Figure 7D). To exhaustively explore the biological functions of these DEGs, we conducted an enrichment analysis using the KEGG database. Among the 478 upregulated DEGs, the significantly enriched pathways were MAPK signaling pathway-plant (ko04016; 21 unigenes); plant-pathogen interaction (ko04626; 20 unigenes); and starch and sucrose metabolism (ko00500; 16 unigenes) (Figure 7E). Among the 1,061 downregulated DEGs, the significantly enriched pathways were ribosome (ko03010; 138 unigenes); phenylpropanoid biosynthesis (ko00940; 36 unigenes); and starch and sucrose metabolism (ko00500; 35 unigenes) (Figure 7F).

For validation of the RNA-Seq data, eight DEGs were examined *via* RT-qPCR (Supplementary Figure 1). The expression patterns were identical between RT-qPCR and RNA-Seq, suggesting that our sequencing data were reliable. These analyses revealed that multiple physiological mechanisms are involved in stamen development after foliar treatment with zebularine, 5-azacytidine, ethrel, and ZT.

### Untargeted Metabolic Profiling by Liquid Chromatography/Mass Spectrometry to Characterize the Combined Treatment Response

**FIGURE 8:**
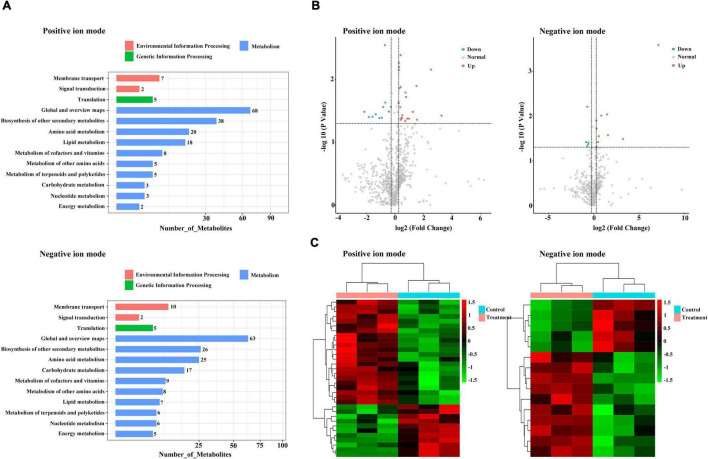
Metabolomic profiles of the treated and control groups. **(A)** KEGG pathways of all annotated metabolites. **(B)** Volcano plot of differentially accumulated metabolites. **(C)** Heatmap of differentially accumulated metabolites.

For metabolic assessment of the effects of DEGs in response to combined treatment with zebularine, 5-azacytidine, ethrel, and ZT, we performed metabolite profiling using untargeted liquid chromatography/mass spectrometry. In total, 790 and 494 metabolites were identified in positive- and negative-ion modes, respectively; 274 and 178 metabolites were annotated to the KEGG database, respectively (Supplementary Tables 6, 7). These annotated KEGG pathways mainly belonged to global and overview maps, as well as biosynthesis of other secondary metabolites (Figure 8A). Among the identified metabolites, 33 (including 22 increased metabolites and 11 decreased metabolites) and 15 (including 10 increased metabolites and 5 decreased metabolites) were significantly altered in the positive- and negative-ion mode, respectively (Supplementary Table 8 and Figure 8B). As indicated by heat maps in Figure 8C, combined treatment with zebularine, 5-azacytidine, ethrel, and ZT altered the levels of numerous metabolites. Among SCMs in the positive-ion mode, 13 increased and 7 decreased metabolites had no KEGG information. The remaining 13 metabolites were annotated to the KEGG database, including noticeable increases in treated samples that were linked to N-benzylformamide; 2-hydroxycinnamic acid; l-isoleucine; 7-methoxycoumarin; 11(z),14(z),17(z)-eicosatrienoic acid; l-tryptophan; skatole; indole-3-lactic acid; and camphor. Decreases in treated samples were linked to 11β-hydroxyandrosterone, anandamide, nefazodone, and ligustilide. Among SCMs in the negative-ion mode, 7 increased and 3 decreased metabolites had no KEGG information; the remaining 5 metabolites were annotated to the KEGG database. Three annotated metabolites were increased in treated samples: uridine monophosphate (UMP), cyclamic acid, and indole-3-lactic acid; two metabolites were decreased in treated samples: ornithine and manidipine.

### Correlation Analysis of Differentially Expressed Metabolites and Transcripts to Characterize the Combined Treatment Response

**FIGURE 9:**
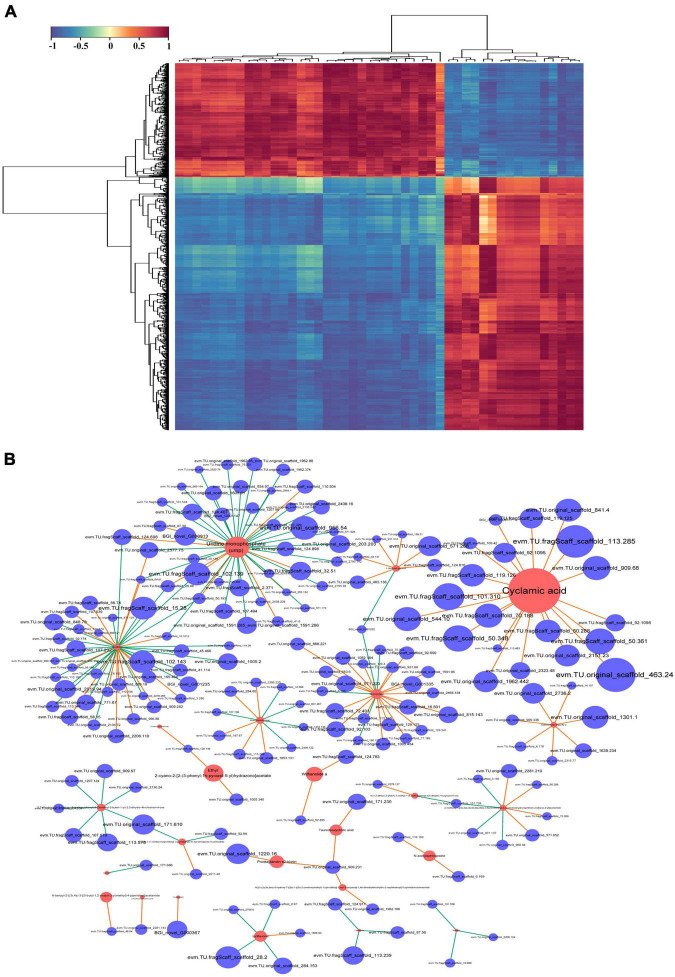
Correlation analysis of differentially expressed metabolites and transcripts. **(A)** Heatmap of differentially accumulated DEGs and significantly changed metabolites (SCMs). **(B)** Network of the 182 DEGs and 24 metabolites with strong correlations. Red circles represent metabolites, blue circles represent genes, and edges represent their relationships. Red and green edges represent positive and negative correlations, respectively, with a Pearson correlation coefficient > 0.99.

**FIGURE 10:**
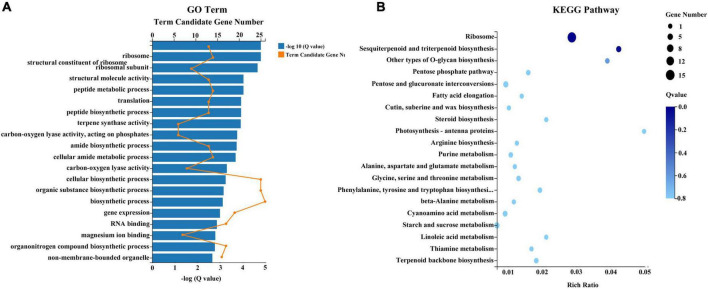
GO **(A)** and KEGG **(B)** pathway enrichment analyses of the 117 DEGs.

To identify key genes and metabolites involved in the response to combined treatment with zebularine, 5-azacytidine, ethrel, and ZT, correlation analyses of metabolites and transcripts were conducted. Pairwise correlation analysis of all DEGs and SCMs is shown in Figure 9A. Notably, 182 DEGs were strongly correlated with 24 metabolites (Figure 9B and Supplementary Table 9). Among these DEGs, two genes were not expressed in treated samples: evm.TU.original_scaffold_1591.285_evm.TU.original_scaffold_1 591.286 and evm.TU.original_scaffold_1301.1; these were negatively correlated with UMP and azithromycin impurity, respectively. In addition, the expression patterns of 42 genes were strongly correlated with UMP; 39 genes were strongly correlated with ornithine; 23 genes were strongly correlated with monoolein; and 20 genes were strongly correlated with cyclamic acid. Subsequently, 117 genes that exhibited strong correlations with metabolites of UMP, ornithine, monoolein, and cyclamic acid were subjected to GO term and KEGG pathway enrichment analyses. The most abundant GO categories were biosynthetic process (GO:0009058; 26 unigenes), cellular biosynthetic process (GO:0044249), and organic substance biosynthetic process (GO:1901576; 25 unigenes) (Figure 10A). The most highly enriched KEGG pathway in the treatment group, compared with the control group, was the ribosome (ko03010; 15 unigenes) (Figure 10B).

## Discussion

To switch from the vegetative state to the reproductive state, flowering plants must coordinate various environmental signals (e.g., photoperiod and temperature) and endogenous stimuli (e.g., phytohormones and carbohydrates) during the life cycle (Zhu et al., 2020). Sexual reproduction is a key process in the plant life cycle; it is strongly influenced by both genetic and environmental conditions (Zhu et al., 2020). Factors such as drought, temperature, salinity, and phytohormones extensively contribute to plant growth and development, inducing various morphological, physiological, and biochemical responses (Utsumi et al., 2019; Li F. et al., 2020). Metabolomics data can connect genomic and phenotypic manifestations (Xia et al., 2021). For plants, survival in different conditions requires substantial metabolic changes, which are reflected by extensive transcriptional level changes that occur during exposure to stress (Janiak et al., 2018). RNA-Seq analysis can provide information regarding the transcriptional regulation of gene expression; this is an effective approach for data mining in species (e.g., persimmon) where genomic data are unavailable (Utsumi et al., 2019; Li Y. S. et al., 2020; Wang L. et al., 2020). Integrative analysis from biological-omics data (i.e., metabolomics combined with transcriptomics) reveals differential metabolites that are related to phenotypic changes, as well as genes that cause changes in such metabolites (Shi et al., 2017; Liu D. et al., 2019; Qiao et al., 2021). In the present study, we investigated the roles of ethrel and ZT in regulating the morphological, cellular, physiological, and molecular characteristics of persimmon flowers. We also investigated the transcriptional and metabolic changes that occurred when the plants were treated with a combination of zebularine, 5-azacytidine, ethrel, and ZT; the findings may be useful for efforts to artificially regulate flower development.

### Effects of Ethrel and Zeatin on Sexual Regulation

Consistent with our previous findings, applications of 1,000 mg/L ethrel and 10 mg/L ZT were sufficient to strengthen the inhibition of male flower development, resulting in androecia developmental inhibition in male flowers. Teixeira et al. (2005) reported that when the mitochondrial structure was disrupted in tissues with a high energy requirement, male sterility occurred. Moreover, cell division was affected by the reduced energy production by mitochondria in flower meristems; this behavior had a negative effect on pollen development (Sabar et al., 2003). Thus, we presume that the enhanced growth of the aborted pistil in samples treated with 10 mg/L ZT was a result of mitochondrial activity, compared with abnormal mitochondrial activity in untreated samples.

Nitrogen is a primary constituent element of protein and nucleic acid; it is vital for increased cell numbers and sizes during flower formation (Ahmed et al., 2017). The altered cell patterns in the aborted pistil, in response to treatment with 10 mg/L ZT, may be related to the increased N content and associated increase in pistil length. In addition, increased amino acid synthesis requires an increased supply of nitrogen (Lu et al., 2017). Thus, the accumulation of total nitrogen presumably contributed to the reduced levels of methionine and cysteine in flowers treated with 1,000 mg/L ethrel and 10 mg/L ZT. Sucrose serves as a major trophic factor and source of energy for axillary bud outgrowth through its enhancement of cellular osmotic activity (Fichtner et al., 2017). Miao et al. (2011) reported that sugar metabolism was involved in the formation of cucumber female flowers in response to low temperature. Here, we found that sucrose was the most accumulated carbohydrate in flowers treated with 10 mg/L ZT, indicating that the increased length of the aborted pistil in male flowers may have been partially induced by an increased sucrose concentration. In addition, the effect of IAA on flowering in *Pharbitis nil* may be mediated by increased ethylene production (Wilmowicz et al., 2014). Similarly, the level of endogenous IAA increased subsequent to the exogenous application of 1,000 mg/L ethrel in this study. A previous study reported that tZ was related to genes responsible for feminization, and the precursor of ZT, tZR, has been identified as an inducer of masculinization in *Mercurialis annua* (Louis et al., 1990). However, we found that the concentrations of endogenous tZ, ZT, and tZR decreased in androecious persimmon flowers following treatment with 1,000 mg/L ethrel, but increased after ZT treatment.

Detecting the corresponding genes showing altered expression would enhance our understanding of the mechanisms underlying plant growth regulator responses. We detected a > 12-fold increase in the expression of *MeGI*, which has been reported as a female growth promoter and male growth inhibitor gene in *Diospyros* (Akagi et al., 2014, 2016), in androecious persimmon flowers treated with 10 mg/L of ZT, demonstrating the inhibition of androecia development by ZT. ETHYLENE RESPONSE FACTORS (*ERFs*) are among the most important transcription factor families, and genes such as *WIN1* and *ERF6* are downstream targets of ethylene signaling (Kannangara et al., 2007; Liu et al., 2016). SMALL AUXIN UP RNA (*SAUR*) gene family members have been widely employed as auxin-inducible reporters, and the *Arabidopsis* genes *SAUR19*–*24* alter phenotypes by increasing plant cell expansion (Spartz et al., 2012). *WIN1*, *ERF6*, and *SAUR22* were highly expressed following exogenous application of 1,000 mg/L of ethrel. Cytokinin dehydrogenase (*CKX*) catalyzes the irreversible degradation of cytokinin in plants and is involved in cytokinin regulatory processes in flower bud development and floral sex differentiation (Cai et al., 2018). DEHYDRATION RESPONSIVE ELEMENT BINDING PROTEIN (*DREB*) family genes were found to be enriched in an ABA-independent pathway (Lata and Prasad, 2011). In the present study, the levels of *CKX6* and *DREB3* in ZT-treated samples were significantly higher than those in control samples, demonstrating their important role in persimmon flower development. *WUSCHEL* (*WUS*) is necessary for female development (Wang et al., 2017) and regulates the behaviors of various types of cells during *Arabidopsis* ovule development (Groß-Hardt et al., 2002). In the present study, we found increased levels of *WUS* expression in androecious persimmon flowers treated with ZT, confirming its role in sexual regulation.

### Effects of Combined Treatment on Sexual Regulation

In addition to ethrel and ZT, zebularine and 5-azacytidine (both of which function as methylation inhibitors; Akagi et al., 2016; Zhou et al., 2016) have been proposed to influence epigenetic regulation of the sex determination process in persimmon. Therefore, we examined phenotypic and microstructural changes in stamens and pollens after exogenous application of 1 mM zebularine + 0.2 mM 5-azacytidine + 2 g/L ethrel + 20 mg/L ZT. Morphological and phenotypic analyses revealed that many shriveled pollen grains with irregular morphology were produced by treated plants, which altered the pollen viability and diameter, while inhibiting stamen development.

To the best of our knowledge, there have not been comprehensive investigations of genes that respond to combined treatment with plant regulators responsible for phenotypic changes in persimmon flowers. Thus, there is a need to identify such genes and related pathways. No significant difference in *MeGI* expression (evm.TU.fragScaff_scaffold_41.132) was detected between the treated and control samples, with FPKM values < 1. We speculate that the combined treatment of ethrel, ZT, zebularine, and 5-azacytidine may affect persimmon flower development mainly by regulating the expression of downstream genes in the *OGI*/*MeGI* system, probably including hormone-related genes or floral organ development-related genes, rather than the expression of *OGI*/*MeGI* itself. Carbon metabolism is essential for plant development through its roles in energy production and the maintenance of cell structure (Hu et al., 2018); pollen absorb soluble sugars and carbohydrates conveyed by the tapetum (Pacini, 1996). We found that 36 upregulated genes were involved in the carbohydrate metabolic process. Of these genes, evm.TU.fragScaff_scaffold_107.249 (homologous to *AMY*) encodes alpha-amylase and was exclusively expressed in the treatment group; this gene was strongly expressed in zebularine + 5-azacytidine + ethrel + ZT-treated flowers, which implies that the inhibited stamen and pollen activities in male flowers were partially related to increased levels of carbohydrate metabolism-related genes. In addition, the mitogen-activated protein kinase (MAPK) cascade is a common signaling mechanism in plants; it comprises a class of conserved modules for the conversion of exogenous stimuli (e.g., mitogens, hormones, developmental signals, pathogens, and other environmental stresses) to molecular and cellular responses (Meng et al., 2014; Kumar et al., 2020). Our results showed that, among upregulated DEGs in treated samples, a significantly enriched KEGG pathway was the MAPK signaling pathway-plant; these findings suggest that the combined treatment with four chemicals (zebularine, 5-AzaC, ethrel, and ZT) led to altered development of stamen and pollen through the expression of genes that are associated with the MAPK signaling pathway. The ribosome is an essential ribonuclear protein complex with roles in plant growth and development that involve translational regulation; ribosomal protein deficiency usually causes growth defects (e.g., reduced shoot growth, reduced cell proliferation, and increased nuclear ploidy in leaf cells) (Byrne, 2009; Rosado et al., 2010; Horiguchi et al., 2011). In the embryo, ribosomal protein gene RPL27aC is required for growth maintenance, as well as the transition from radial to bilateral symmetry associated with cotyledon initiation (Szakonyi and Byrne, 2011a). In addition, abnormal apical-basal patterning of the gynoecium is found in multiple ribosomal protein mutants (Nemhauser et al., 2000; Szakonyi and Byrne, 2011b). Thus, the 138 downregulated DEGs enriched in the ribosome pathway presumably contributed to the male flower defects in treated samples.

The integration of related genes and metabolites provides molecular insights into persimmon flower development after combined treatment with zebularine, 5-azacytidine, ethrel, and ZT. Coexpression analysis involving genes and metabolites showed that, of the 182 DEGs that exhibited distinct correlations with 24 metabolites, more than half (i.e., 117 genes) had strongly positive or negative correlations with UMP, ornithine, monoolein, and cyclamic acid; these findings suggest that these genes may have important regulatory roles in the ribosome pathway. Notably, UMP and cyclamic acid exhibited the greatest change among the 24 SCMs; both of them were accumulated in the treated samples. UMP is an intracellular nucleotide that serves as a nitrogen source (Kim et al., 1999). In plant cells, pyrimidines are derived from UMP, which is formed by either *de novo* synthesis or salvaging of preformed nucleobases or nucleosides (Stasolla et al., 2003). Most of the correlated genes exhibited negative relationships with UMP, including one gene that was absent from treated samples: evm.TU.original_scaffold_1591.285_evm.TU.original_scaffold_1 591.286, which encodes glutathione S-transferase and was annotated to the glutathione metabolism KEGG pathway. Increased synthesis of amino acids requires an increased supply of nitrogen (Lu et al., 2017), which is a primary constituent element of protein and nucleic acid; nitrogen is vital for increased cell numbers and sizes during flower formation (Ahmed et al., 2017). Our results imply that UMP has a strong association with stamen development. Furthermore, cyclamic acid functions as a type of artificial sweetener (Leban et al., 2007); the positive expression levels of 20 significantly altered genes indicated that they promoted the accumulation of cyclamic acid. In summary, coexpression analysis of UMP and cyclamic acid metabolite-related genes in the ribosome pathway provided insights into persimmon sex differentiation after combined treatment with zebularine, 5-azacytidine, ethrel, and ZT; the mechanism underlying this regulation requires further investigation. However, an intriguing phenomenon was observed during sampling: the treated samples were extremely fragile and could not be physically handled by the research team. Thus, there is a need to identify appropriate treatment timing and exogenous substance concentrations, as well as synergistic and antagonistic effects among hormones; these factors are essential for successful control of flower phenotypes.

## Conclusion

**FIGURE 11:**
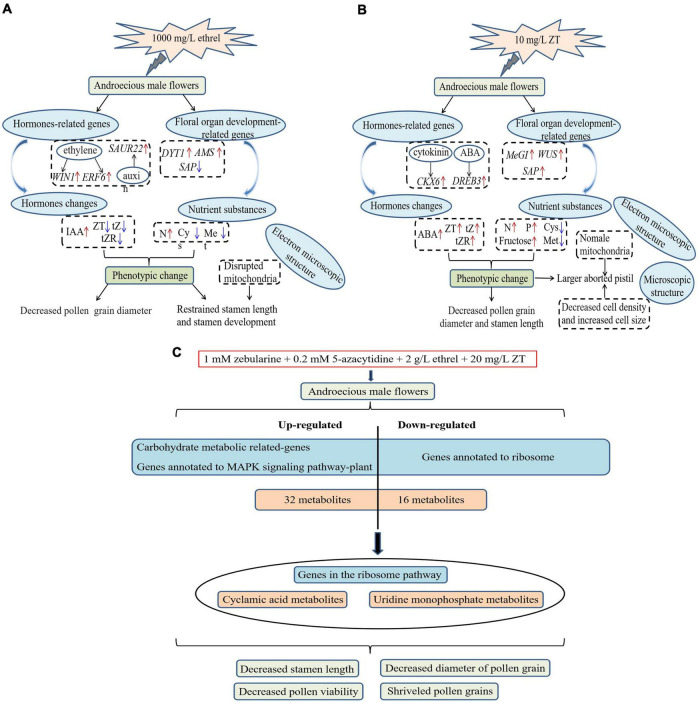
Proposed regulatory networks activated in persimmon flowers in response to exogenous chemicals. **(A)** Treatment with 1000 mg/L ethrel. **(B)** Treatment with 10 mg/L ZT. **(C)** Combined treatment with zebularine, 5-azacytidine, ethrel, and ZT. Expression levels are shown using a color gradient from low (blue) to high (red).

In this study, all persimmon trees exhibited robust responses to treatments with exogenous reagents (Figure 11). Ethrel at 1,000 mg/L and ZT at 10 mg/L both significantly reduced the stamen length and pollen grain diameter in androecious trees; treatment with 10 mg/L ZT also led to arrested pistil development. These phenotypic changes were consistent with altered carbohydrate, amino acid, and endogenous phytohormone contents, as well as changes in hormones and floral organ development-associated genes, further demonstrating the inhibitory effects of ZT and ethrel on male flower development. In addition, we investigated the effects of combined treatment with zebularine, 5-azacytidine, ethrel, and ZT on androecious persimmon. Morphological and phenotypic comparisons revealed that stamen length, pollen viability, and pollen grain diameter were significantly inhibited. Transcriptomic analyses identified 1,539 DEGs that were primarily involved in carbohydrate metabolism and MAPK signaling pathway-plant processes; these were also annotated to the ribosome pathway. Furthermore, metabolome analysis revealed 33 and 15 metabolites in positive- and negative-ion modes, respectively. Overall, the data indicate that 182 DEGs were strongly correlated with 24 metabolites; the integration of important genes in the ribosome pathway provides molecular insights into the regulation of UMP and cyclamic acid metabolites. Our findings provide an important basis for future efforts to control sex differentiation in flowers.

## Data Availability Statement

The datasets presented in this study can be found in online repositories. The names of the repository/repositories and accession number(s) can be found below: https://ngdc.cncb.ac.cn/gsa/s/3ZC6458B, CRA006019.

## Author Contributions

PS, FL, and JF conceived and designed the experiments. LW and HL carried out the experiments. LW wrote the manuscript. YS, WH, SD, YM, YW, JY, LY, TP, QZ, and FL helped to draft and review the manuscript. All authors read and approved the final manuscript.

## Conflict of Interest

The authors declare that the research was conducted in the absence of any commercial or financial relationships that could be construed as a potential conflict of interest.

## Publisher’s Note

All claims expressed in this article are solely those of the authors and do not necessarily represent those of their affiliated organizations, or those of the publisher, the editors and the reviewers. Any product that may be evaluated in this article, or claim that may be made by its manufacturer, is not guaranteed or endorsed by the publisher.

## Footnotes

1
https://github.com/trinityrnaseq

2
http://www.bioconductor.org/packages/release/bioc/html/DESeq2.html

## References

[B1] Ahmed R. Ahmed S. Karim M. R. Siddiky M. A. (2017). Effect of N, P and K fertilizer on the flower yield of *Chrysanthemum*. *Agriculturists* 15 58–67. 10.3329/agric.v15i1.33429

[B2] Akagi T. Henry I. M. Kawai T. Comai L. Tao R. (2016). Epigenetic regulation of the sex determination gene *MeGI* in polyploid persimmon. *Plant Cell* 28 2905–2915. 10.1105/tpc.16.00532 27956470 PMC5240738

[B3] Akagi T. Henry I. M. Ohtani H. Morimoto T. Beppu K. Kataoka I. (2018a). A Y-encoded suppressor of feminization arose *via* lineage-specific duplication of a cytokinin response regulator in Kiwifruit. *Plant Cell* 30 780–795. 10.1105/tpc.17.00787 29626069 PMC5969274

[B4] Akagi T. Pilkington S. M. Varkonyi-Gasic E. Henry I. M. Sugano S. S. (2018b). Two Y-chromosome-encoded genes determine sex in kiwifruit. . *Nat. Plants* 5 801–809. 10.1038/s41477-019-0489-6 31383971

[B5] Akagi T. Henry I. M. Tao R. Comai L. (2014). A Y-chromosome-encoded small RNA acts as a sex determinant in persimmons. *Science* 346 646–650. 10.1126/science.1257225 25359977

[B6] Akagi T. Katayama-Ikegami A. Yonemori K. (2011). Proanthocyanidin biosynthesis of persimmon (*Diospyros kaki* Thunb.) fruit. *Sci. Hortic.* 30 373–380. 10.1016/j.scienta.2011.07.021

[B7] Byrne M. E. (2009). A role for the ribosome in development. *Trends Plant Sci.* 14 512–519. 10.1016/j.tplants.2009.06.009 19716746

[B8] Cai L. Zhsang L. Fu Q. T. Xu Z. F. (2018). Identification and expression analysis of cytokinin metabolic genes IPTs, CYP735A and CKXs in the biofuel plant *Jatropha curcas*. *PeerJ.* 6:e4812. 10.7717/peerj.4812 29785355 PMC5960259

[B9] Ferguson A. C. Pearce S. Band L. R. Yang C. Ferjentsikova I. King J. (2017). Biphasic regulation of the transcription factor Aborted Microspores (AMS) is essential for tapetum and pollen development in Arabidopsis. *New Phytol.* 213:778. 10.1111/nph.14200 27787905 PMC5215365

[B10] Fichtner F. Barbier F. F. Feil R. Watanabe M. Annunziata M. G. Chabikwa T. G. (2017). Trehalose 6-phosphate is involved in triggering axillary bud outgrowth in garden pea (*Pisum sativum* L.). *Plant J.* 92 611–623. 10.1111/tpj.13705 28869799

[B11] George A. P. Mowat A. D. Collins R. J. Morley-Bunker M. (1997). The pattern and control of reproductive development in nonastringent persimmon (*Diospyros kaki* L.): a review. *Sci. Hortic.* 70 93–122. 10.1016/s0304-4238(97)00043-5

[B12] Groß-Hardt R. Lenhard M. Laux T. (2002). WUSCHEL signaling functions in interregional communication during *Arabidopsis* ovule development. *Genes Dev.* 16 1129–1138. 10.1101/gad.225202 12000795 PMC186242

[B13] Gu J. N. Zhu J. Yu Y. Teng X. D. Lou Y. Xu X. F. (2014). DYT1 directly regulates the expression of *TDF1* for tapetum development and pollen wall formation in Arabidopsis. *Plant J.* 80:1005. 10.1111/tpj.12694 25284309

[B14] Guan C. F. Zhang P. X. Wu M. H. Zeng M. Chachar S. Ruan X. F. (2020). Discovery of a millennial androecious germplasm and its potential in persimmon (*Diospyros kaki* Thunb.) breeding. *Sci. Hortic.* 269:109392. 10.1016/j.scienta.2020.109392

[B15] Harkess A. Huang K. Hulst R. (2020). Sex determination by two Y-linked genes in garden Asparagus. *Plant Cell* 32 1790–1796. 10.1105/tpc.19.00859 32220850 PMC7268802

[B16] Hartwig T. Chuck G. S. Fujioka S. Klempien A. Weizbauer R. Potluri D. P. V. (2011). Brassinosteroid control of sex determination in maize. *Proc. Natl. Acad. Sci. U.S.A.* 108 19814–19819. 10.1073/pnas.1108359108 22106275 PMC3241820

[B17] Horiguchi G. Mollá-Morales A. Pérez-Pérez J. M. Kojima K. Robles P. Ponce M. R. (2011). Differential contributions of ribosomal protein genes to *Arabidopsis thaliana* leaf development. *Plant J.* 65 724–736. 10.1111/j.1365-313X.2010.04457.x 21251100

[B18] Hu L. Xie Y. Fan S. J. Wang Z. S. Wang F. H. Zhang B. (2018). Comparative analysis of root transcriptome profiles between drought tolerant and susceptible wheat genotypes in response to water stress. *Plant Sci.* 272 276–293. 10.1016/j.plantsci.2018.03.036 29807601

[B19] Huang Y. F. Luo Z. R. Zhang Q. L. (2013). Phylogenetic analysis of some androecious genotypes native to China and related *Diospyros spp*. using chloroplast fragments. *Acta Hortic.* 996 103–109. 10.17660/actahortic.2013.996.11 34854763

[B20] Janiak A. Kwasniewski M. Sowa M. Gajek K. Zmuda K. Kościelniak J. (2018). No time to waste: transcriptome study reveals that drought tolerance in barley may be attributed to stressed-like expression patterns that exist before the occurrence of stress. *Front. Plant Sci.* 8:2212. 10.3389/fpls.2017.02212 29375595 PMC5767312

[B21] Kannangara R. Branigan C. Liu Y. Penfifield Y. Rao V. Mouille G. (2007). The transcription factor WIN1/SHN1 regulates cutin biosynthesis in *Arabidopsis thaliana*. *Plant Cell* 19 1278–1294. 10.1105/tpc.106.047076 17449808 PMC1913754

[B22] Kim M. K. Lee I. Y. Ko J. H. Rhee Y. H. Park Y. H. (1999). Higher intracellular levels of uridinemonophosphate under nitrogen-limited conditions enhance metabolic flux of curdlan synthesis in *Agrobacterium* species. *Biotechnol. Bioeng.* 62 317–323. 10.1002/(sici)1097-0290(19990205)62:3<317::aid-bit8>3.0.co;2-7 10099543

[B23] Kondo H. Shiraya T. Wada K. C. Takeno K. (2010). Induction of flowering by DNA demethylation in *Perilla frutescens* and *Silene armeria*: heritability of 5-azacytidine-induced effects and alteration of the DNA methylation state by photoperiodic conditions. *Plant Sci.* 178 321–326. 10.1016/j.plantsci.2010.01.012

[B24] Kumar K. Raina S. K. Sultan S. M. (2020). *Arabidopsis* MAPK signaling pathways and their cross talks in abiotic stress response. *J. Plant Biochem. Biotechnol.* 29 700–714. 10.1007/s13562-020-00596-3

[B25] Lata C. Prasad M. (2011). Role of DREBs in regulation of abiotic stress responses in plants. *J. Exp. Bot.* 62, 4731–4748. 10.1093/jxb/err210 21737415

[B26] Leban I. Tasic D. R. Lah N. Klofutar C. (2007). Structures of artificial sweeteners - cyclamic acid and sodium cyclamate with other cyclamates. *Acta Cryst. B* 63 418–425. 10.1107/S0108768107013961 17507755

[B27] Lee D. H. Lee I. C. Kim K. J. Kim D. S. Na H. J. Lee I. (2014). Expression of gibberellin 2-oxidase 4 from Arabidopsis under the control of a senescence-associated promoter results in a dominant semi-dwarf plant with normal flowering. *J. Plant Biol.* 57 106–116. 10.1007/s12374-013-0528-1

[B28] Li F. Chen X. Yu X. Chen M. Lu W. Wu Y. (2020). Novel insights into the effect of drought stress on the development of root and caryopsis in barley. *PeerJ* 8:e8469. 10.7717/peerj.8469 32030325 PMC6996498

[B29] Li Y. S. Wang X. R. Li Y. Zhang Y. J. Gou Z. W. Qi X. S. (2020). Transcriptomic analysis revealed the common and divergent responses of maize seedling leaves to cold and heat stresses. *Genes* 11:881. 10.3390/genes11080881 32756433 PMC7464670

[B30] Li S. Z. Sun P. Du G. G. Wang L. Y. Li H. W. Fu J. M. (2019). Transcriptome sequencing and comparative analysis between male and female floral buds of the persimmon (*Diospyros kaki* Thunb.). *Sci. Hortic.* 246 987–997. 10.1016/j.scienta.2018.11.073

[B31] Liu D. Cheng Y. Z. Gong M. Zhao Q. Jiang C. H. Cheng L. R. (2019). Comparative transcriptome analysis reveals differential gene expression in resistant and susceptible tobacco cultivars in response to infection by cucumber mosaic virus. *Crop J.* 307–321. 10.1016/j.cj.2018.11.008

[B32] Liu W. J. Huang S. N. Liu Z. Y. Lou T. X. Tan C. Wang Y. H. (2019). A missense mutation of STERILE APETALA leads to female sterility in Chinese cabbage (*Brassica campestris* ssp. *pekinensis*). *Plant Reprod.* 32 217–228. 10.1007/s00497-019-00368-7 30806770

[B33] Liu M. Gomes B. L. Mila I. Purgatto E. Peres L. E. P. Frasse P. (2016). Comprehensive profifiling of ethylene response factor expression identifies ripening-associated ERF genes and their link to key regulators of fruit ripening in tomato. *Plant Physiol.* 170 1732–1744. 10.1104/pp.15.01859 26739234 PMC4775140

[B34] Liu R. Liu Y. G. Ye N. H. Zhu G. H. Chen M. X. Jia L. G. (2015). AtDsPTP1 acts as a negative regulator in osmotic stress signalling during *Arabidopsis* seed germination and seedling establishment. *J. Exp. Bot.* 66 1339–1353. 10.1093/jxb/eru484 25540435 PMC4339596

[B35] Louis J. P. Augur A. Teller G. (1990). Cytokinins and differentiation processes in *Mercurialis annua*. *Plant Physiol.* 94 1535–1541. 10.1104/pp.94.4.1535 16667886 PMC1077417

[B36] Lu J. M. Yang R. T. Wang H. C. Huang X. M. (2017). Stress effects of chlorate on longan (*Dimocarpus longan* Lour.) trees: changes in nitrogen and carbon nutrition. *Hortic. Plant J.* 3 237–246. 10.1016/j.hpj.2017.12.003

[B37] Luo Z. R. Wang R. Z. (2008). Persimmon in China: domestication and traditional utilizations of genetic resources. *Adv. Hortic. Sci.* 22 239–243.

[B38] Martin A. Troadec C. Boualem A. Rajab M. Fernandez R. Morin H. (2009). A transposon-induced epigenetic change leads to sex determination in melon. *Nature* 461 1135–1138. 10.1038/nature08498 19847267

[B39] Massonnet M. Cochetel N. Minio A. (2020). The genetic basis of sex determination in grapes. *Nat. Commun.* 11:2902. 10.1038/s41467-020-16700-z 32518223 PMC7283251

[B40] Mateo-Bonmatí E. Casanova-Sáez R. Ljung K. (2019). Epigenetic regulation of auxin homeostasis. *Biomolecules* 9:623. 10.3390/biom9100623 31635281 PMC6843323

[B41] Meng Y. L. Ma N. Zhang Q. You Q. Li N. Khan M. (2014). Precise spatio-temporal modulation of ACC synthase by MPK6 cascade mediates the response of rose flowers to rehydration. *Plant J.* 79 941–950. 10.1111/tpj.12594 24942184

[B42] Miao M. Yang X. Han X. Wang K. (2011). Sugar signalling is involved in the sex expression response of monoecious cucumber to low temperature. *J. Exp. Bot.* 62 797–804. 10.1093/jxb/erq315 20937729

[B43] Müller N. A. Kersten B. Leite Montalvão A. P Mähler N. Bernhardsson C. Bräutigam K. (2018). A single gene underlies the dynamic evolution of poplar sex determination. *Nat. Plants* 6 630–637. 10.1038/s41477-020-0672-9 32483326

[B44] Nemhauser J. L. Feldman L. J. Zambryski P. C. (2000). Auxin and ETTIN in *Arabidopsis* gynoecium morphogenesis. *Development* 127 3877–3888. 10.1242/dev.127.18.3877 10952886

[B45] Pacini E. (1996). Types and meaning of pollen carbohydrate reserves. *Sex. Plant Reprod.* 9, 362–366. 10.1007/BF02441957

[B46] Pawełkowicz M. E. Skarzyńska A. Pląder W. Przybecki Z. (2019). Genetic and molecular bases of cucumber (*Cucumis sativus* L.) sex determination. *Mol. Breed.* 39:50.

[B47] Plackett R. G. A. Thomas G. S. Wilson A. Z. Hedden P. (2011). Gibberellin control of stamen development: a fertile field. *Trends Plant Sci.* 16 568–578. 10.1016/j.tplants.2011.06.007 21824801

[B48] Qiao Y. Cheng Q. M. Zhang Y. T. Yan W. Yi F. Y. Shi F. L. (2021). Transcriptomic and chemical analyses to identify candidate genes involved in color variation of sainfoin flowers. *BMC Plant Biol.* 21:61. 10.1186/s12870-021-02827-8 33482728 PMC7825240

[B49] Rosado A. Sohn E. J. Drakakaki G. Pan S. Swidergal A. Xiong Y. (2010). Auxin-mediated ribosomal biogenesis regulates vacuolar trafficking in Arabidopsis. *Plant Cell* 22 143–158. 10.1105/tpc.109.068320 20061553 PMC2828701

[B50] Sabar M. Dominique G. Balk J. Leaver C. J. (2003). ORFB is a subunit of F1F0-ATP synthase: insight into the basis of cytoplasmic male sterility in sunflower. *EMBO Rep.* 4 381–386. 10.1038/sj.embor.embor800 12671689 PMC1319156

[B51] Saito S. Fujii N. Miyazawa Y. Yamasaki S. Matsuura S. Mizusawa H. (2007). Correlation between development of female flower buds and expression of the *CS-ACS2* gene in cucumber plants. *J. Exp. Bot.* 58 2897–2907. 10.1093/jxb/erm141 17630291

[B52] Shi J. Yan B. Y. Lou X. P. Ma H. S. Ruan S. L. (2017). Comparative transcriptome analysis reveals the transcriptional alterations in heat-resistant and heat-sensitive sweet maize (*Zea mays* L.) varieties under heat stress. *BMC Plant Biol.* 17:26. 10.1186/s12870-017-0973-y 28122503 PMC5267381

[B53] Spartz A. K. Lee S. H. Wenger J. P. Gonzalez N. Itoh H. Inze D. (2012). The SAUR19 subfamily of SMALL AUXIN UP RNA genes promote cell expansion. *Plant J.* 70 978–990. 10.1111/j.1365-313X.2012.04946.x 22348445 PMC3481998

[B54] Stasolla C. Katahira R. Thorpe T. A. Ashihara H. (2003). Purine and pyrimidine nucleotide metabolism in higher plants. *J. Plant Physiol.* 160 1271–1295. 10.1078/0176-1617-01169 14658380

[B55] Sun P. Li J. R. Du G. G. Han W. J. Fu J. M. Diao S. F. (2017). Endogenous phytohormone profiles in male and female floral buds of the persimmons (*Diospyros kaki* Thunb.) during development. *Sci. Hortic.* 218 213–221. 10.1016/j.scienta.2017.02.022

[B56] Szakonyi D. Byrne M. E. (2011a). Ribosomal protein L27a is required for growth and patterning in *Arabidopsis thaliana*. *Plant J.* 65 269–281. 10.1111/j.1365-313X.2010.04422.x 21223391

[B57] Szakonyi D. Byrne M. E. (2011b). Involvement of ribosomal protein RPL27a in meristem activity and organ development. *Plant Signal. Behav.* 6 712–714. 10.4161/psb.6.5.15070 21448008 PMC3172845

[B58] Teixeira R. T. Farbos I. Glimelius K. (2005). Expression levels of meristem identity and homeotic genes are modified by nuclear-mitochondrial interactions in alloplasmic male-sterile lines of *Brassica napus*. *Plant Cell* 42 731–742. 10.1111/j.1365-313X.2005.02407.x 15918886

[B59] Trivellini A. Ferrante A. Vernieri P. Serra G. (2011). Effects of abscisic acid on ethylene biosynthesis and perception in *Hibiscus rosa-sinensis* L. flower development. *J. Exp. Bot.* 62 5437–5452. 10.1093/jxb/err218 21841180 PMC3223042

[B60] Utsumi Y. Utsumi C. Tanaka M. Ha C. V. Takahashi S. Matsui A. (2019). Acetic acid treatment enhances drought avoidance in cassava (*Manihot esculenta* Crantz). *Front. Plant Sci.* 10:521. 10.3389/fpls.2019.00521 31105723 PMC6492040

[B61] Wang L. Y. Li H. W. Sun P. Suo Y. J. Han W. J. Diao S. F. (2021). Effects of plant growth regulators, soil moisture contents and carbon/nitrogen ratios on sex differentiation in persimmon (*Diospyros kaki* Thunb.) flowers. *J. Plant Growth Regul.* 40 1121–1138. 10.1007/s00344-020-10170-9

[B62] Wang J. Tian C. H. Zhang C. Shi B. H. Cao X. W. Zhang T. Q. (2017). Cytokinin signaling activates WUSCHEL expression during axillary meristem initiation. *Plant Cell* 29 1373–1387. 10.1105/tpc.16.00579 28576845 PMC5502442

[B63] Wang L. Y. Li H. W. Sun P. Fu J. M. Suo Y. J. Zhang J. J. (2018). Genetic diversity among wild androecious germplasms of *Diospyros kaki* in China based on SSR markers. *Sci. Hortic.* 242 1–9. 10.1016/j.scienta.2018.07.020

[B64] Wang L. Y. Li H. W. Suo Y. J. Han W. J. Diao S. F. Mai Y. N. (2020). Programmed cell death facilitates the formation of unisexual male and female flowers in persimmon (*Diospyros kaki* Thunb.). *Agronomy* 10:234. 10.3390/agronomy10020234

[B65] Wang L. Zheng B. Yuan Y. Xu Q. L. Chen P. (2020). Transcriptome profiling of *Fagopyrum tataricum* leaves in response to lead stress. *BMC Plant Biol.* 20:54. 10.1186/s12870-020-2265-1 32013882 PMC6998078

[B66] West N. W. Golenberg E. M. (2018). Gender-specific expression of GIBBERELLIC ACID INSENSITIVE is critical for unisexual organ initiation in dioecious *Spinacia oleracea*. *New Phytol.* 217 1322–1334. 10.1111/nph.14919 29226967

[B67] Wilmowicz E. Frankowski K. Kucko A. Kesy J. Kopcewicz J. (2014). Involvement of the IAA-regulated ACC oxidase gene *PnACO3* in Pharbitis nil flower inhibition. *Acta Biol. Crac. Ser. Bot.* 56 90–96. 10.2478/abcsb-2014-0013

[B68] Xia Y. Chen W. W. Xiang W. B. Wang D. Xue B. G. Liu X. Y. (2021). Integrated metabolic profiling and transcriptome analysis of pigment accumulation in *Lonicera japonica* flower petals during colour-transition. *BMC Plant Biol.* 21:98. 10.1186/s12870-021-02877-y 33596836 PMC7890969

[B69] Xu J. C. Zhang Q. L. Xu L. Q. Guo D. L. Luo Z. R. (2016). Recent developments in deastringency mechanism of persimmon fruit. *Acta Hortic. Sin.* 43 1653–1664.

[B70] Yakushiji H. Ymada M. Yonenori K. Sato A. Kimura N. (1995). Staminate flower production on shoots of ‘Fuyu’ and ‘Jiro’ persimmon (*Diospyros kaki* Thunb.). *J. Jpn. Soc. Hortic. Sci.* 64 41–46. 10.2503/jjshs.64.41

[B71] Zhang P. X. Yang S. C. Liu Y. F. Zhang Q. L. Xu L. Q. Luo Z. R. (2016). Validation of a male-linked gene locus (*OGI*) for sex identification in persimmon (*Diospyros kaki* Thunb.) and its application in F1 progeny. *Plant Breed.* 135 721–727. 10.1111/pbr.12427

[B72] Zhou H. Liao J. Liu B. Azam M. Xia Y. P. (2016). Effects of 5-azacytidine and gibberellic acid on flower development of azalea. *Pak. J. Agri. Sci.* 53 1–6.

[B73] Zhu L. Guan Y. X. Liu Y. N. Zhang Z. H. Jaffar M. A. Song A. P. (2020). Regulation of flowering time in chrysanthemum by the R2R3 MYB transcription factor CmMYB2 is associated with changes in gibberellin metabolism. *Hortic. Res.* 7:96. 10.1038/s41438-020-0317-1 32637124 PMC7326907

