# Supplementary material for: Effects of Different Chemicals on Sexual Regulation in Persimmon (Diospyros kaki Thunb.) Flowers
Source: Front Plant Sci. 2022 May 26;13:876086. doi: 10.3389/fpls.2022.876086 (PMC9179176; doi:10.3389/fpls.2022.876086)
Supplement: Supplementary file 1 [file Data_Sheet_1.docx]

Supplementary Material

# 1 Supplementary Figure


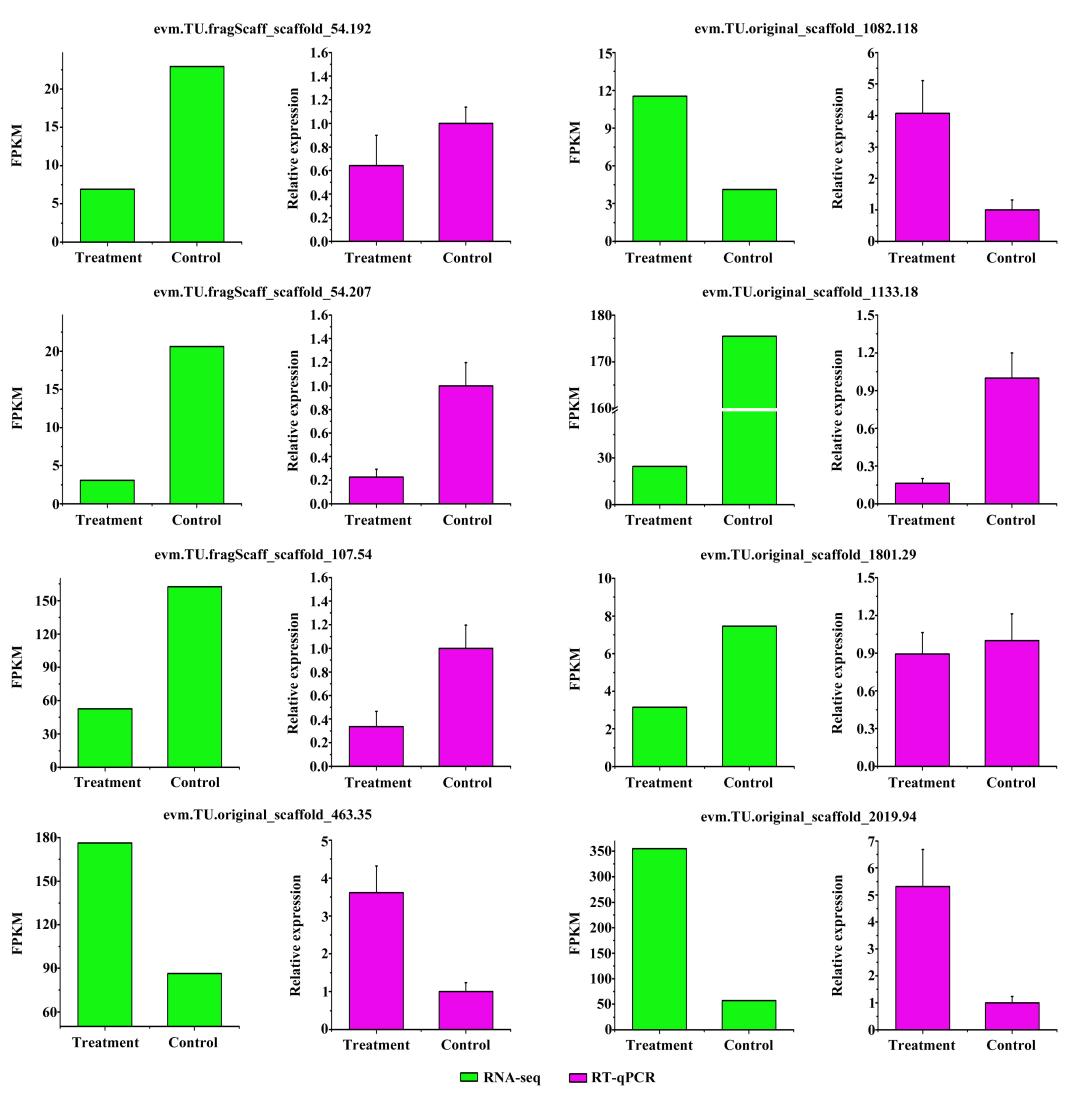


**Supplementary Figure 1** RT-qPCR validation of differential transcription predicted by RNA-seq.

# 2 Supplementary Tables

**Supplementary Table 1** Sequences of primers used for the genes analyzed for ethrel and ZT treatment.

| **Gene ID**  **(Homologous Gene)** | **Gene Function** | **Primer Sequence（5’-3’）** | **Size of PCR Fragment (bp)** |
| --- | --- | --- | --- |
| *GAPDH* | Reference gene | F: AGCTCTTCCACCTCTCCAGT  R: TGCTAGCTGCACAACCAACT | 157 |
| *MeGI*  (c100711_g1) | Female growth promoter and male growth inhibitor gene in *Diospyros* (Akagi et al., 2014, 2016). | F: GGAGTTGAACTTTGGGAACG  R: AAGGCGACACTTGTGGACGA | 199 |
| *DREB3*  (c97305_g1) | A gene induced by abiotic stress and involved in the ABA-independent pathway (Lata and Prasad, 2011). | F: CCCAGTTTACATAGGAGTGCGAA  R: GGTGGGGGAAGTTGAGAATGG | 182 |
| *WIN1*  (c90561_g1) | Downstream target gene of ethylene signaling (Kannangara et al., 2007). | F: ATCAACATCACTCATCGGCTGC  R: GGAGGAGAAAACGACCACACAAAT | 152 |
| *ERF6*  (c114128_g2) | Downstream target gene of ethylene signaling (Liu et al., 2016). | F: TCAAACCGACCTAACAATACCGA  R: AACTCGGTTCGCTACTGGAAATAA | 157 |
| *DYT1*  (c100018_g1) | A gene that regulates tapetum and pollen development (Gu et al., 2014). | F: ACCTGTCTCCGTTTCTCTCCGA  R: TTGGATCTGTAGCCTGAAGCCT | 150 |
| *GA20OX2*  (c105233_g1) | A gene promoting GA biosynthesis (Lee et al., 2014). | F: GGATGAGCCTGGGAGTAAG  R: TGTGCCTAAGGTCTGGTCT | 116 |
| *WUS*  (c109151_g3) | A gene that regulates female development (Wang et al., 2017). | F: AGGTAGAATCTGAGAACCCGCC  R: GAAACCCTTCCTCTCTTCCCC | 184 |
| *AMS*  (c121208_g1) | A gene that regulates tapetum and pollen development (Ferguson et al., 2017). | F: GTTATGGTGATGGTGGCGTCCT  R: GATAACGCGGAATCCAGCCA | 121 |
| *SAP*  (c105715_g1) | A gene that leads to female sterility (Liu et al., 2019b). | F: TATGGGATAGCCAAACCGAACA  R: CTCCCACAAGACGAATCCCTG | 203 |
| *CKX6*  (c118893_g1) | A gene involving in cytokinin regulatory process (Cai et al., 2018). | F: CTGTCAAACGCAGGGATTAGTG  R: ATTCTTGCTCGGGTGATTATGC | 185 |
| *CYP707A4*  (c65956_g1) | ABA-catabolism gene (Liu et al., 2015). | F: GATGCGAACAGAACTCCTACGA  R: AGATAATCAGCGAAACGAGGGA | 139 |
| *SAUR22*  (c98330_g1) | A gene employed as auxin-inducible reporter (Spartz et al., 2012). | F: TTCTTGTTATTGGTCCCACGGT  R: CGAAAAAAATGCTTTACAGGCG | 151 |

**Supplementary Table 2** The primers of genes used in RT-qPCR analyses for mixture of zebularine, 5-azacytidine, ethrel, and ZT treatment.

| **Gene ID** | **Forward Sequence (5’-3’)** | **Reverse Sequence (5’-3’)** | **Size of PCR Fragment (bp)** |
| --- | --- | --- | --- |
| *GAPDH* | AGCTCTTCCACCTCTCCAGT | TGCTAGCTGCACAACCAACT | 157 |
| evm.TU.fragScaff_scaffold_54.192 | ATGGCAGCAGGAGACAGTGG | ACAAGGGCCAGGCAGAGTTT | 152 |
| evm.TU.original_scaffold_1082.118 | CATACACCGGCGAGCACAAC | ATCGCTGATGGCCATGTCCA | 209 |
| evm.TU.fragScaff_scaffold_54.207 | TAGAGGTGGGCTACGTTGCG | GCCGGTGAGCTTCCTGATCT | 152 |
| evm.TU.original_scaffold_1133.18 | TGGCTTCCAAGAGCAATGCT | TTGGATGAGGCTGCAGCATG | 239 |
| evm.TU.fragScaff_scaffold_107.54 | TATGCCCGTGAGCTCCCTTG | ATGGCCTCCTATCTGCTGGC | 184 |
| evm.TU.original_scaffold_1801.29 | TACCCACCCACAACAGCTCG | ATTCCCTCAACCCCTCTGCG | 173 |
| evm.TU.original_scaffold_463.35 | ACGGCAGCTAACCTCTTCCC | ATGGAGGGCTCGACTCAAGC | 178 |
| evm.TU.original_scaffold_2019.94 | GCCATTGACGAAGAGGCTGC | GCGTAGTACAGCGGCGTTTC | 225 |

**Supplementary Table 3** Output quality data for the six sequencing libraries.

| **Sample** | **Total Raw Reads (M)** | **Total Clean Reads (M)** | **Total Clean Bases (Gb)** | **Clean Reads Q20 (%)** | **Clean Reads Q30 (%)** | **Clean Reads Ratio (%)** |
| --- | --- | --- | --- | --- | --- | --- |
| Control-1 | 45.57 | 44.55 | 6.68 | 97.42 | 92.97 | 97.76 |
| Control-2 | 45.57 | 44.72 | 6.71 | 97.31 | 92.68 | 98.13 |
| Control-3 | 45.57 | 44.5 | 6.67 | 97.38 | 92.92 | 97.64 |
| Treatment-1 | 45.57 | 44.05 | 6.61 | 97.29 | 92.7 | 96.66 |
| Treatment-2 | 45.57 | 44.11 | 6.62 | 97.49 | 93.24 | 96.78 |
| Treatment-3 | 45.57 | 45.06 | 6.76 | 97.23 | 92.47 | 98.87 |

Q20: Percentage of bases with a Phred value > 20

Q30: Percentage of bases with a Phred value > 30
